# Impact of the COVID-19 Pandemic on the Urologist’s clinical practice in Brazil: a management guideline proposal for low- and middle-income countries during the crisis period

**DOI:** 10.1590/S1677-5538.IBJU.2020.04.03

**Published:** 2020-05-20

**Authors:** Arie Carneiro, Marcelo Langer Wroclawski, Bruno Nahar, Andrey Soares, Ana Paula Cardoso, Nam Jin Kim, Fabricio Torres Carvalho

**Affiliations:** 1 Departamento de Urologia Hospital Albert Einstein São PauloSP Brasil Departamento de Urologia , Hospital Albert Einstein , São Paulo , SP , Brasil ;; 2 Grupo Internacional de Urologia Avançada São PauloSP Brasil Diretor Científico e Executivo - Grupo Internacional de Urologia Avançada , São Paulo , SP , Brasil ;; 3 Departamento de Urologia Beneficiência Portuguesa de São Paulo São PauloSP Brasil Departamento de Urologia - Beneficiência Portuguesa de São Paulo , São Paulo , SP , Brasil ;; 4 Department of Urology University of Miami Miller School of Medicine FL USA Department of Urology , University of Miami Miller School of Medicine , FL , USA ;; 5 Departamento de Oncologia Médica Hospital Albert Einstein São PauloSP Brasil Departamento de Oncologia Médica , Hospital Albert Einstein , São Paulo , SP , Brasil ;; 6 Departamento de Oncologia Médica Centro Paulista de Oncologia São PauloSP Brasil Departamento de Oncologia Médica , Centro Paulista de Oncologia – Oncoclínicas, São Paulo , SP , Brasil ;; 7 Grupo Latino-Americano de Oncologia Cooperativa São PauloSP Brasil Diretor científico - Grupo Latino-Americano de Oncologia Cooperativa , São Paulo , SP , Brasil ;; 8 Programa de Cirurgia e Cirurgia Robótica Hospital Albert Einstein São PauloSP Brasil Chefe do Programa de Cirurgia e Cirurgia Robótica , Hospital Albert Einstein , São Paulo , SP , Brasil ;; 9 Departamento de Doenças Infecciosas Hospital Albert Einstein São PauloSP Brasil Departamento de Doenças Infecciosas , Hospital Albert Einstein , São Paulo , SP , Brasil ;; 10 Departamento de Medicina Intensiva Unidade de Terapia Intensiva AC Camargo Cancer Center São PauloSP Brasil Departamento de Medicina Intensiva e Unidade de Terapia Intensiva - AC Camargo Cancer Center , São Paulo , SP , Brasil

## Abstract

This letter to the Editor aims to provide suggestions and recommendations for the management of urological conditions in times of COVID-19 crisis in Brazil and other low- and middle-income countries. It is important to highlight that one of the main characteristics of this pandemic is the oversaturation of the health system capacity, mostly due to a high demand for personal protective equipment (PPE), Hospital/ICU beds, as well as ventilators. In places with limited resources and where the health care systems are already saturated, such consideration is even more worrisome. Therefore, most worldwide authorities are recommending to avoid, as much as possible, patient’s elective visits to hospitals, as well as a judicious use of the operating room in order to mitigate the strain put on the health system. While efforts should be directed to the care of COVID-19 patients, other conditions (especially urgencies and oncological cases) must continue to be assisted. Thus, through a panel of experts, we have prepared a practical guide for urologists based on the recommendations from the main Urologic Associations, as well as data from the literature to support the suggested management. We will try to follow the standard guideline recommendations from the American Urological Association (AUA) and European Association of Urology (EAU), with the aim of pursuing the best outcomes possible. However, some recommendations were based on the consensus of the panel, taking into consideration the reality of developing countries and the unprecedented situation caused by the COVID-19 crisis.

Most importantly, all recommendations on this manuscript are based on the expectancy of a maximum 3-month duration of the crisis. If this period shall extended, these recommendations will be revised and updated.

The format of the text will be given through questions and answers.

## How much is the pandemic by COVID-19 impacting the clinical practice of the urologist?

Similar to other specialties, the pandemic has drastically changed the routine of the urologists. Elective clinic visits are being canceled, postponed or, in some situations, replaced by remote care through telemedicine, recently regulated and temporarily authorized by the Brazilian Ministry of Health ( 1 ).

We believe that tele-screening, test reviews and follow-up evaluations that do not require physical examination are the ideal situations for this type of care, especially when the patient is in the high-risk group and must be socially isolated ( 2 ).

In regard to surgeries, all postponable procedures must be rescheduled, in order to reduce the exposure of the surgical team and the patient to a potential contamination. Furthermore, cancelation of surgeries collaborates with social isolation and save resources (such as PPEs) for the care of patients with COVID-19 infection. The main question is how to define which operations can really be postponed, especially in urologic oncology, without interfering with the patient’s outcomes.

## What general care should be taken in any type of surgery during this period?

The most important recommendation at this point is that elective surgical procedures should be postponed. The diagnostic, therapeutic and human resources of the Health Care facilities must be available to fight the pandemic( 3 ). Some considerations should be made:

- We must consider all cases as suspect, until proven otherwise. Ideally, every case should be tested by rRT-RNA-CRP for SARS-CoV-2 48 prior to surgery, but unfortunately this is not feasible in most developing countries. Negative confirmed cases should be kept in a separate environments.
- Surgeries for COVID-19 negative patients should ideally be performed in a surgical center different from the location where patients with positive COVID-19 are being treated. If it is not possible to separate an entire surgical block, we suggest designating specific rooms for the care of patients with COVID-19 that will not be used for regular cases.
- A trained and dedicated multidisciplinary team should be available for the management of suspected and confirmed patients for COVID-19. It is preferable that this team does not assist COVID-19 negative cases.
- Whenever possible, we should prioritize surgeries with local anesthesia or spinal blockade.
- Always obtain a consent form, as recommended by the Brazilian Society of Medical and Bioethics Law. Patients are at risk of contracting COVID-19 infection during their hospital stay and major surgeries in asymptomatic infected patients during the incubation period appear to predict worse outcomes, with a mortality rate up to 20% ( 3 ).
- After the procedure, COVID-19 positive patients should be admitted to the designated areas for suspected and / or confirmed patients with COVID-19, if the institution in question provides such area.

## Should we always perform pre-surgical screening?

- If available, we recommend testing all patients for rRT-RNA-PCR for SARS-CoV-2 48 hours before performing the procedure.
- If it is impossible to test everyone with the resources available, all cases should be considered suspect.

## In case of surgery, what is the proper vestment and PPEs for health care providers?

For everyone in the room: caps, personal protective glasses, N95 mask (PFF2 or PFF3), protective gowns for contacts, procedure gloves and shoe covers. For those who will perform procedures: cap, personal protective glasses, face shield, N95 mask (PFF2 or PFF3), sterile waterproof apron, sterile gloves, shoe covers and waterproof disposable boots whenever secretions (when urine, stool or blood are expected, such as in endourological procedures) are expected.

- In the setting of N95 masks rationing, the face shield is important in order to prevent soiling of the mask, that can be further reutilized.
- Increased care should be taken when handling patient’s stool. Studies have shown that even in patients with negative airway CRP, the clearance of the the viral RNA is longer in the stool ( 4 ). Thus, surgeons should take extra precautions with surgeries that include bowel manipulation and trans-rectal prostate biopsies.

## What is the correct way of surgical vesting in COVID-19 positive cases?

a) On the corridor:

- Hand Hygiene;
- Put on the N95 mask and goggles or face shield for anesthetists, in the case of intubation.
- Perform surgical hand antisepsis.

b) In the lobby

- Put on a surgical gown.
- Put on sterile gloves (Surgical team);
- The anesthesiologist should use 2 pairs of gloves and after intubation the second pair should be changed as soon as possible.

## What is the correct order to remove the surgical PPE?

a) Inside the room:

- Remove gloves;
- Hand hygiene;
- Remove disposable gown;
- Hand hygiene.

b) Outside the room: (Leave a side table with an Oxivir® drum and procedure gloves).

- Remove glasses;
- Remove the N95 mask and place it in an identified plastic bag;
- Remove the cap;
- Hand hygiene;
- Put on procedure gloves and clean and disinfect the glasses and support surface (Use disinfectant detergent - Oxivir® or Optigerm ®);
- Remove the gloves;
- Hand hygiene. After removing protective equipment, remember not to touch your hair or face before hand washing.

## What special care should we take in laparoscopic / robotic surgery?

Some studies have suggested transmission of some pathogens (coryneobacterium, papillomavirus and HIV) by the pneumo peritoneum through the release of smoke generated by the laparoscopic electrocautery ( 5 - 7 ). A parallel situation may be extrapolated to the coronavirus.

Therefore, additional care in these procedures must be performed:

- As mentioned earlier, it is important to test all patients before the procedure, if possible.
- Additional protection in relation to aerosol dispersion: Always keep materials clean, assistants must have additional care when placing and removing trocars, do not use trocars with air leakage, avoid using monopolar energy and give preference to bipolar, keep settings of the electrocautery to minimum in order to reduce smoke formation.
- Handling of pneumoperitoneum: keep it as low as possible, minimizing the Trendelenburg as much as possible. If possible, use devices that are able to aspirate and filtrate the smoke from the pneumoperitoneum.
- During disinflation, the CO2 gas and smoke should be captured with an ultra-filtration system. A disinflation mode should be used on your insufflator if available.
- If available, use filters on vacuum cleaners, there are different models and brands.
- Use drains only when extremely necessary because post-operative care in the presence of organic fluids demand extra-caution and additional PPE.
- Favor the open approach in cases where minimally invasive surgery has not shown considerable benefit.

## What general care should urologists take during the pandemic?

### When should we go into isolation?

Urologists, like other physicians, should be isolated only when they become suspected or confirmed cases of COVID-19. In suspicious cases, the SARS-CoV-2 RT-RNA-CRP should be collected and physicians should be kept isolated until the result.

Physicians with the following symptoms should be considered highly suspicious for COVID-19: fever, respiratory symptoms (cough, runny nose, nasal obstruction, sore throat, shortness of breath, loss of smell), in addition to body aches, fatigue, diarrhea and nausea ( 8 ).

### When should we perform the test?

Healthcare professionals should always be tested when symptomatic.

### How long is quarantine recommended?

The duration of the quarantine is 14 days, starting on the day of onset of symptoms. Individuals should be asymptomatic at the end of this period. Otherwise, they should remain isolated until symptoms are resolved, and only return to activities 72 hours after the resolution of all symptoms. The Center for Disease Control and Prevention (CDC-USA) recommends the utilization of RT-RNA-CRP for control and only release physicians to work after a negative result. However, due to the lack of tests in most of the country, this is not mandatory by the Brazilian Ministry of Health ( 9 ).

## In general urology, which surgeries should not be postponed?

### In cases of patients with urinary lithiasis

All surgeries to treat urolithiasis should be suspended, unless these are emergencies.

In the presence of ureteral lithiasis associated with fever or other signs of infection, there is an absolute indication for antibiotic therapy and urinary drainage. Preferably, we opted for the passage of a ureteral stent (i.e double J stent) under spinal anesthesia (or even with local anesthesia). As an alternative, bedside ultrasound percutaneous guided nephrostomy might be considered in centers with the necessary expertise( 10 ).

In addition to lithiasis associated with urinary tract infection, ureteral obstruction in a solitary kidney or bilateral ureteral obstruction, acute impairment of renal function and pain refractory to clinical treatment should not be postponed. Unlike other recommendations( 11 ), our position is that, once the surgical procedure is indicated, we should be as resolutive as possible, in order to reduce the number of visits to the hospital for new surgeries to the emergency department. Thus, instead of just draining the urinary tract, our tendency is to perform ureterolithotripsy whenever possible and safe, keeping the double J stent with a string externalized by the urethra to be removed on an outpatient basis.

The remaining cases of renal colic should preferably be managed clinically, with medical expulsive therapy and pain control. However, it is important to note that, invariably, cases initially conducted in this way may evolve into emergency situations, such as those previously mentioned.

Patients who are already with a double J stent may remain with the stent for as long as possible. Surgery may be indicated in cases of extreme ureteral stent discomfort. Otherwise, clinicians must keep strict control of all cases in order to avoid the forgotten double J syndrome.

### In cases of patients with benign prostatic hyperplasia

Patients with benign prostatic hyperplasia should not be operated at this time of a pandemic, unless they develop complication that will require hospitalization and possible surgery, such as massive hematuria and clot retention. In this scenario, we believe that the evacuation of clots and / or cauterization of the prostate should already be accompanied by resection, vaporization or endoscopic enucleation of the prostate ( 12 ).

In all other cases, even if there is urinary retention, we recommend postponing the procedure. If necessary, indwelling urinary catheter placement or percutaneous cystostomy with local anesthesia are indicated for preservation of renal function( 13 , 14 ).

### In cases of patients with hematuria

The investigation of hematuria through imaging tests during COVID-19 pandemic should be limited to cases of macroscopic hematuria, especially if there are clots or hemoglobin decrease. Greater attention should be given to patients at higher risk for urothelial carcinoma, such as men, over 50 years of age and with a history of smoking and exposure to known carcinogenic agents.

The gold standard test for investigation of the upper urinary tract is uro-tomography, but in times when we need to consider the use of resources, ultrasound could potentially be used since many imaging services are overloaded due to the frequent indication of thoracic CTs for the diagnosis and follow-up of patients with Sars-Cov-2. These data are extrapolations from recent evidence that ultrasound could replace tomography in the investigation of microscopic hematuria ( 14 ). However, once the outbreak is resolved or if the resource is available, uro-tomography should be performed.

Regarding the lower urinary tract, flexible cystoscopes are not widely avaliable in Brazil. Thus, diagnostic cystoscopies should initially be postponed avoiding hospitalization for cystoscopy in the OR. Ultrasound could also be used to evaluate the lower urinary tract during this time of pandemic.

### In cases of urological emergencies

In addition to the procedures previously mentioned, non-urologic oncology conditions that deserve urgent treatment are testicular torsion, scrotal abscess and / or Fournier’s Syndrome, infection of penile prosthesis or artificial sphincter, priapism and urological trauma. We should consider postponing surgical treatment for all other urological conditions, such as urinary incontinence, prolapses, urethral stenosis, prosthetic implants, infertility-related operations (including vasectomy) and genital procedures such as circumcision or hydrocele correction ( 15 ).

## Outpatient procedures how should we proceed?

### Urodynamic study:

We suggest that urodynamic study programs should be suspended during the crisis period.

### Surveillance and follow-up cystoscopy:

Whenever possible, cystoscopies should be postponed. If indispensable, priority should be given to outpatient procedures, using a flexible cystoscope.

It’s well known that most programs in developing countries do not have flexible cystoscopy, so it is necessary to perform it in the operating room. This should be postponed whenever possible.

### Prostate biopsy

As a rule, prostate biopsies should be postponed, since delaying the diagnosis of prostate cancer for 3-6 months will not interfere with survival outcomes in the vast majority of cases. It’s worth discussing prostate biopsy in highly suspicious cases of patients with symptoms related to advanced / metastatic disease, such as bone pain or urinary retention. Given that most medications for metastatic prostate cancer (such as androgen deprivation therapy) are not approved/released without histological confirmation of prostate adenocarcinoma, we recommend to biopsy the most easily accessible site, which may be the prostate (often the most quickly available resource) or some metastasis focus.

However, if clinical signs of metastatic disease are evident, a shared decision should be made with the patient and additional efforts should be performed to expedite the release of these medications even without the biopsy. We believe this exception should be highly considered in selected patients, especially in times of crisis.

If indicated, the biopsy should be performed under local prostatic block, avoiding sedation. As mentioned earlier, additional care must be taken, given the high prevalence of COVID-19 in the stool of infected patients.

### Intravesical instillation

- In case of small bladder tumors, consider a single-dose intravesical chemotherapy within 24 hours of TURBT (not immunotherapy). The most commonly used agents are: mitomycin and gemcitabine, in Brazil just gemcitabine isavaiable ( 16 ).
- In Intermediate-risk and high-risk non-muscle-invasive bladder cancers: Clinically fit patients with no major comorbidities should receive induction therapy followed by at least 1-year maintenance BCG. In selected cases we can consider postpone one dose during the maintenance. In the case of BCG shortage supply, gemcitabine can be used( 16 , 17 ).

## How should we manage genitourinary cancers during COVID-19 pandemic?

### Patients with D’Amico low-risk prostate cancer

- Treatment: Active Surveillance is recommended for all patients with Grade Group 1.
- Follow-up: Follow-up tests as well as confirmatory and control biopsies should be postponed.

### Patients with D’Amico’s Intermediate Risk Prostate Cancer

- Treatment: Treatment of these patients should be postponed until the pandemic is over. We have robust evidence to support that postponing treatment in these patients for 3 months do not impact cancer-specific mortality (PROTECT, PIVOT, SPCG 4 trials). Afterward, local treatment should follow the current recommendations in the guidelines ( 18 - 20 ).
- Follow-up: Patients who have already been treated should ideally be followed via telehealth. In-person consultations should only be carried out if they are really necessary. The first post-operative PSA can be performed after 3 months, because no early adjuvant therapy would be initiated in this scenario.

### Patients with D’Amico high-risk prostate cancer

- Treatment: We recommend initiation of hormone deprivation therapy immediately and, after 3 months, discuss the most appropriate local therapy. There is good evidence supporting this approach, particularly when associated with radiation therapy and, less common, as neoadjuvant for surgery. While no survival benefit was seen with neoadjuvant studies, pre-operative androgen deprivation therapy reduced positive margin rates as well as extra prostatic extension without compromising cancer control ( 21 ).
- Follow-up: Patients who have already been treated should undergo additional tests and visit after the pandemic. In-person consultations should only be carried out if they are really necessary. The first post-operative PSA can be performed after 3 months, because no early adjuvant therapy would be initiated in this scenario.

### Biochemical recurrence

No adjuvant radiation therapy may be indicated during COVID-19 pandemic. All cases, even in the presence of unfavorable features, can be managed later with salvage radiation therapy if necessary.

## Metastatic prostate cancer

### Patients with castration-sensitive disease

We recommend use of ADT in a 6-month formulation ( 22 ) in association with Apalutamide 240mg VO daily or Enzalutamide 160mg VO daily ( 23 ), when indicated.

If Apalutamide or Enzalutamide is not available, an alternative option is abiraterone 1000mg VO daily associated with prednisone 5mg VO daily. The use of low dose prednisone should be considered since the impact of corticoid usage during the COVID-19 pandemic are not well known ( 24 ).

Chemotherapy associated with ADT should be indicated only in extremely selected cases. When indicated, it can be postponed until 120 days after initiation of ADT ( 25 ).

Use of colony stimulating factor is recommended in cases of chemotherapy.

### Patients with castration-resistant disease

We recommend the use of ADT in semiannual formulation ( 22 ) associated with preferably 160mg of enzalutamide VO daily (if not previously received) ( 26 ) or alternatively abiraterone 1000mg VO daily associated with prednisone 5mg VO daily (if not previously received).

Alternatively, in patients with isolated bone metastases, the use of radium 223 ADT may be considered ( 27 ).

Alternatively, docetaxel with reduced dose every 3 weeks can be considered. It’s important to emphasize that colony stimulating factor is highly recommended when chemotherapy is administered ( 28 ). Zoledronic acid should be used in patients with bone metastases every 3 months ( 29 ).

### Localized kidney neoplasm

Asymptomatic cT1a patients should have their treatment postponed, unless there is a rare risk that a nephron-sparing procedure becomes not feasible with the delay of surgery.

Asymptomatic cT1b or cT2 patients, eligible to partial nephrectomy, should be operated to avoid losing the window of a nephron sparing surgery. If the indication is radical nephrectomy, it may be postponed.

Patients with cT3-4 disease and/or with symptoms such as gross hematuria should be operated, especially those with thrombus in the renal vein and / or vena cava.

## Metastatic kidney neoplasm

### What is the role of cytoreductive nephrectomy in the current scenario? Should we always try systemic treatment first?

We should proceed with cytoreductive nephrectomy whenever this is the best short- and medium-term treatment option for symptoms control. Asymptomatic patients with low and intermediate risk who can wait 3 months to start treatment should wait. Patients with indications of immediate systemic treatment should start treatment despite nephrectomy, especially in cases of intermediate and poor risk ( 30 ). Regarding the choice of first line treatment, we favor, if available, the combination of a tyrosine kinase inhibitors (TKI) with immunotherapy, such as Axitinib with pembrolizumab ( 31 , 32 ).

We also suggest adjusting the dose intervals to less frequent (pembrolizumab to 400mg every 6 weeks). If not available, the choice between the combination of immunotherapy with ipilimumab and nivolumab versus TKI should take into account the risk of complications and potential readmissions ( 33 ).

### Systemic treatment

It is relatively safe to start systemic therapy with tyrosine kinase inhibitors (TKI) and immunotherapy, although it might have a hypothetical detrimental effect on the immunological response to COVID-19. Nonetheless, no reliable evidence regarding therapy with immunotherapy or tyrosine kinase inhibitors has been identified but the landscape is changing rapidly, and we should be attentive to any evidence that could show the opposite ( 30 ).

### Non-invasive bladder neoplasm

In general, TURBT should be performed whenever possible. It is important to highlight that we recommend performing a cold-cup biopsy of the base of the lesion to ensure representation of the detrusor muscle and avoid the need for repeat TURBT due to undersampling ( 16 ).

Elderly patients with multiple comorbidities and asymptomatic patients with radiologically small and superficial tumors can have the procedure postponed. viRADS staging system may play an important role in selecting such cases.

Repeat TURBT is the standard of care for non-muscle-invasive high-risk bladder tumors ( 34 ). However, during the pandemic, some exceptions can be considered. If the initial procedure was performed by an experienced surgeon who is confident that the entire lesion was completely removed and there is presence of muscle layer in the pathology, the repeat TURBT may be postponed. In patients with high-grade pTa, repeat TURBT can also be delayed, even if no muscle is represented.

### Invasive muscle bladder neoplasm

Muscle invasive bladder cancer is an aggressive disease with great potential to be curable. In this scenario, surgical delays might represent a loss on the window of treatment.

In general, we recommend performing neoadjuvant chemotherapy and, after finishing, assess the conditions of the hospital to decide on the need for a cystectomy vs bladder preservation protocol (if indicated). Of note, neoadjuvant chemotherapy with cisplatin and gemcitabine before cystectomy can be delayed for up to 6-8 weeks from diagnosis ( 35 ).

Cystectomy after neoadjuvant chemotherapy can also be safely delayed for up to 10 weeks post chemotherapy ( 36 ) without jeopardizing oncological outcomes ( 37 ).

Patients who have undergone cystectomy should consider adjuvant chemotherapy with cisplatin and gemcitabine. This approach still offers survival benefits even 180 days after surgery ( 38 ). We should always consider using colony stimulating factor when chemotherapy is recommended.

For patients who wish to preserve the bladder, or who are not eligible for surgery, or the hospital does not have adequate resources, trimodally therapy should be considered. (TURBT followed by hypo-fractionated radiotherapy associated with weekly chemotherapy with gemcitabine 100mg/m ^2^ ).

### Metastatic bladder neoplasm

For cisplatin eligible patients we recommend first line of treatment ( 35 ):

- Cisplatin 35mg/m ^2^ on days 1 and 8 and gemcitabine 1000mg/m ^2^ on days 1 and 8 every 3 weeks ( 39 ).
- Consider colony stimulating factor in all patients.

First line treatment for patient’s cisplatin-inegible

For cisplatin ineligible PD-L1 positive patients we recommend first line of treatment:

- Atezolizumab 1200mg on day 1 every 3 weeks ( 40 ) or pembrolizumab 200mg on day 1 every 3 weeks ( 40 ).

For cisplatin ineligible PD-L1 negative patients we recommend the first line of treatment:

- Carboplatin, AUC 4.5-5 and gemcitabine 1,000mg/m ^2^ on day 1 and day 8, every 3 weeks ( 41 ).
- Consider colony stimulating factor in all patients.

### Testicular cancer (initial diagnosis)

Radical orchiectomy should be performed as soon as possible because it is an outpatient procedure and will guide further treatment. We recommend surveillance for most patients with Stage I over any adjuvant treatment, despite unfavorable features ( 42 ).

### Stage 2 Testicular cancer

- For low volume stage II patients (IIa and IIb) we recommend radiotherapy instead of chemotherapy for seminomas ( 42 ).
- For High volume Stage II seminomas: 4 cycles of EP(Etoposide 100mg/m ^2^ IV on Days 1-5 and Cisplatin 20mg/m ^2^ IV on Days 1-5, every 21 days), considering using G-CSF ( 42 );
- For Non-seminoma stage IIA with normal markers: retroperitoneal lymph node dissection might be considered to avoid use of chemotherapy ( 42 ).
- For non-seminoma Stage IIA with significant elevation of tumor markers and stage III (for both seminoma and non-seminoma): In case of favorable risk, we recommend 4 cycles of EP. We do not recommend 3 cycles of BEP. In case of intermediate or unfavorable risk, 4 cycles of VIP (Etoposide 75mg/m ^2^ IV on Days 1-5; Ifosfamide 1200mg/m ^2^ IV on Days 1-5 with same protection and Cisplatin 20mg/m ^2^ IV on Days 1-5, every 21 days) are preferable or alternatively, 4 cycles of BEP, (with strong recommendations of G-CSF use), because of the bleomycin pulmonary toxicity ( 42 ).

## Recommended Reading and updating web sites for healthcare professionals

- Centers for Disease Control and Prevention (CDC). Coronavirus (COVID-19). Available at. <https://www.coronavirus.gov/>
- World Health Organization (WHO). Coronavirus disease (COVID-19) Pandemic. Available at. <https://www.who.int/emergencies/diseases/novel-coronavirus-2019>
- Journal of the American Medical Association (JAMA). Coronavirus Disease 2019 (COVID-19). Available at. <https://jamanetwork.com/journals/jama/pages/coronavirus-alert>
- The New England Journal of Medicine (NEJM). Coronavirus (COVID-19). Available at. <https://www.nejm.org/coronavirus>
- The Lancet (Lancet). COVID-19 Resource Centre. Available at. <https://www.thelancet.com/coronavirus>
